# SEOM-GEIS Spanish clinical guidelines for the management of soft‑tissue sarcomas (2024)

**DOI:** 10.1007/s12094-024-03842-5

**Published:** 2025-02-07

**Authors:** César Serrano, Marta Arregui, Irene Carrasco, Nadia Hindi, Javier Martínez-Trufero, Jerónimo Martínez-García, Áurea Molina, Ana Paisán, Raúl Sánchez, María Ángeles Sala

**Affiliations:** 1 Servicio de Oncología Médica. Hospital, Universitario Vall d’Hebron, Vall d’Hebron Barcelona Hospital Campus, C/ Natzaret, 115-117, 08035 Barcelona, Spain; 2 Servicio de Oncología Médica. Hospital General, Universitario Gregorio Marañón, Madrid, Spain; 3 Servicio de Oncología Médica Hospital, Universitario Virgen del Rocío, Seville, Spain; 4 Servicio de Oncología Médica Fundación Jiménez Díaz, Madrid, Spain; 5 Servicio de Oncología Médica Hospital Universitario Miguel Servet, Saragossa, Spain; 6 Servicio de Oncología Médica Hospital, Universitario Virgen de La Arrixaca, Murcia, Spain; 7 Servicio de Oncología Médica Complejo Hospitalario Universitario de La Coruña, La Coruña, Spain; 8 Servicio de Oncología Médica Hospital Universitario Donostia, San Sebastián, Spain; 9 Servicio de Oncología Médica Hospital Universitario Son Espases, Palma, Spain; 10 Servicio de Oncología Médica Hospital Universitario Basurto, Bilbao, Spain

**Keywords:** Sarcoma, Soft-tissue sarcoma, Guidelines, Rare cancer

## Abstract

Soft-tissue sarcomas are rare, diverse malignant tumors of mesenchymal origin, requiring diagnosis and treatment by a specialized multidisciplinary team. Initial assessment includes radiology and biopsy, followed by wide surgical resection with clear margins for localized cases. Radiotherapy is recommended for large, deep, high-grade tumors or after incomplete resection, while perioperative chemotherapy may be considered for high-risk cases. In oligometastatic disease, combining local and systemic therapies is an option. Anthracycline-based chemotherapy is the first-line treatment in advanced disease, though other drugs show efficacy in certain subtypes. Given the limited options, enrolling in clinical trials is advised for patients needing further treatment.

## Guidelines

### Incidence and epidemiology

Sarcoma constitutes a group of rare and heterogenous malignant neoplasms of mesenchymal origin that includes over 80 different histological entities displaying disease-specific biology, natural history and prognosis. Sarcomas in the adult population are classified into soft tissue sarcomas (STS) (75%), visceral sarcomas (15%), and bone sarcomas (10%). The annual incidence of STS—the objective of this guidelines—in Europe is 4–5 cases/100,000. There is no gender predisposition and, albeit more common in middle-aged and older individuals, STS also affects children and young adults. STS can arise in any part of the body but affects more frequently the extremities [1].

Although STS are largely spontaneous, several genetic syndromes increase their predisposition. Neurofibromatosis type 1 increases the likelihood of developing malignant peripheral nerve sheath tumors (MPNSTs) and gastrointestinal stromal tumors (GISTs). Similarly, SMARCB1-related schwannomatosis increases the likelihood of developing MPNSTs. Patients with Li Fraumeni syndrome, through heritable TP53 mutations, have a 15–20% lifetime risk of STS, and also predisposes to osteosarcoma and other non-mesenchymal neoplasms. Gardner’s syndrome is a variant of the Familial Adenomatous Polyposis (FAP), caused by germline mutations in the adenomatous polyposis coli (APC) gene. In addition to the FAP-related outgrowth of adenomatous polyps, these patients are at risk of developing desmoid tumors, which occur in up to 16% of the cases. Hereditary retinoblastomas are associated with an increased risk for the development of STS, most typically leiomyosarcomas. More recently, heritable defects involving centrosome genes and the sheltering complex have been linked with increased STS propensity [2]. Overall, individuals carrying any of these genetic conditions must be referred to genetic counselling (**III,A**).

Therapeutic irradiation is the most common environmental factor associated with the development of specific sarcoma subtypes (undifferentiated pleomorphic sarcoma, angiosarcoma and leiomyosarcoma) within the irradiated field. Radiation-induced sarcomas have a worse outcome than sporadic sarcomas [3]. Additionally, iatrogenic lymphoedema has been associated with cutaneous angiosarcoma (Stewart-Treves syndrome), and UV radiation with cutaneous angiosarcomas and atypical fibroxanthoma/pleomorphic dermal sarcomas.

### Methodology

This guideline is based on a systematic review of relevant published studies and with the consensus of ten sarcoma-expert oncologists from GEIS (Spanish Group For Sarcoma Research) and SEOM (Spanish Society of Medical Oncology), and an external review panel of two experts designated by SEOM. The Infectious Diseases Society of America-US Public Health Service Grading System for Ranking Recommendations in Clinical Guidelines has been used to assign levels of evidence and grades of recommendation [4].

### Diagnosis, pathology and molecular biology

The recommendation for conducting specific imaging studies and tumor biopsy and their specific sequence must be made within a multidisciplinary committee. The goals of imaging studies are to establish tumor size, depth, site, resectability, and the presence of metastases. Magnetic resonance (MRI) with contrast is the preferred technic for tumors arising in the limbs, pelvis, and trunk wall. For abdominal or retroperitoneal lesions, and for staging purposes, a computed tomography (CT) scan is recommended (**II,A**). FDG-PET/CT-scan be used selectively to solve specific diagnostic challenges, but not as a standard tool during the work-up.

Multiple image-guided core needle biopsies performed by an expert are essential for diagnosis. The biopsy must take into consideration the surgical approach. Incisional and excisional biopsy may be considered only in selected cases (superficial lesions < 3 cm) (**II,A**). Pathological diagnosis should be made according to the most recent WHO classification and histological grading using the FNCLCC system, when applicable (**III,A**) [1, 5]. Biopsies may underestimate the malignancy grade. For cases diagnosed outside of a reference center/network, validation by a pathology expert is strongly recommended (**III,A**) [1]. Diagnosis should be complemented by molecular pathology (FISH, RT-PCR or NGS) (**III,A**), especially when: (1) The specific pathological diagnosis is uncertain; (2) The clinical pathological presentation is unusual; (3) It could be of prognostic and/or predictive significance; (4) The entity specifically points to a unique molecular abnormality; (5) There is evidence of therapeutic targets. Oncologists and pathologists have to verify that the most relevant genes and fusions are included in the molecular panel and that it follows independent quality assessments (Table 1).

**Table 1:** Checklist for diagnosis

Imaging check-list	Pathology check-list at diagnosis	Pathology check-list of the surgical specimen
MRI: gold standard for STS in extremities	Core-needle biopsy performed in a reference center and considering the surgical approach	whether the tumor was intact and the status of surgical margins - R0: Macroscopic complete resection - R1: margins are microscopic involved - R2: Macroscopic incomplete resection
CT extension study according to the STS subtype	Expert validation diagnosis from not expert center	Residual viable tumor cells and mitotic index
Brain CT/MRI: alveolar soft tissue sarcoma, clear cell sarcoma and angiosarcoma	Localization, size and depth Presence or absence of invasion of adjacent structures	Percentage of post-treatment changes: - Necrosis - Sclerohyalinosis - Fibrosis - Fibrohistiocystic reaction - Hemorrhage
Bone scan, whole body MRI are optional in selected subtypes	Histological diagnosis (WHO classification) and grade (FFNCLCC)	Confirmation of pre-surgical diagnosis and evaluation of grade (FFNCLCC)
Regional lymph node metastasis evaluation in selected subtypes	Immunohistochemistry and molecular evaluation	Biobanking

Biobanking of fresh snap-frozen tumor tissue is encouraged in all cases, given the rarity of these diseases and the need to foster further research (**V,A**).

#### Management of STS patients in reference centers

As a general principle, both diagnosis and treatment decision should be performed in a coordinated and structured manner at sarcoma-expert centers (in Spain, Centros, Servicios y Unidades de Referencia – CSUR) and/or within reference networks with multidisciplinary expertise and high patient volumes (**III,A**). It is well demonstrated that the management of STS patients within these centers and networks improve their outcomes [6].

Recommendations:

Both diagnosis and treatment decisions of a clinical suspicion of sarcoma should be conducted in a sarcoma-expert center within a multidisciplinary committee (**III,A**).

### Staging and risk assessment

The UICC TNM 8th edition [7] is the most widely used staging system for STS. Other prognostic factors include anatomic site, age, histological subtype, and the quality of surgical margins. Clinical nomograms are also available to estimate prognosis and can be of help to individualize treatment decisions, especially in the neo/adjuvant setting [8].

The most common pattern of spread is hematogenous, with pulmonary metastases being the most frequent, and, therefore, a chest CT scan is recommended for staging in all cases (**II,A**). Regional lymph node involvement is rare (< 1%), but more frequent in certain subtypes such as rhabdomyosarcoma, epithelioid sarcoma, clear cell sarcoma, angiosarcoma, and synovial sarcoma. Regional assessment with CT/MRI should be considered for these tumors. Additionally, a CT scan of the abdomen is recommended for myxoid liposarcoma, leiomyosarcoma, epithelioid sarcoma, and angiosarcoma. Brain imaging should be considered for alveolar soft part sarcoma, clear cell sarcoma, and angiosarcoma. In myxoid liposarcoma, an MRI of the spine can be considered (Table 1).

Recommendation:

CT and/or MRI contrast-enhanced followed by core needle biopsy are the gold standard methods for STS diagnosis (**II,A**).

### Management of local and loco-regional disease

**Fig. 1:**
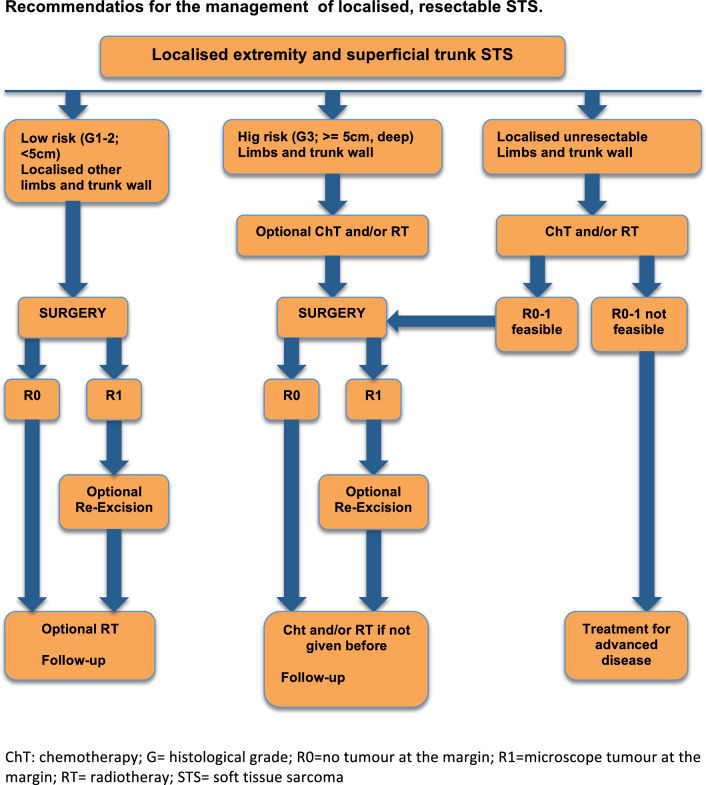
Algorithm summarizing the recommendations for the management of localized, resectable soft tissue sarcomas from extremities and trunk wall

The management of localized disease is displayed in the algorithm in Fig. 1.

#### Surgery and radiotherapy

Surgery is the standard treatment for localized STS. The preferred surgical procedure consists of a complete *en bloc* tumor resection with negative margins (R0) (**II,A**) [9]. The required negative margins depend on tumor location, histology, grade, and preoperative therapies, although at least 1 cm or an intact anatomical barrier (fascia, adventitia, periosteum, or epineurium) is recommended. The biopsy tract should be resected along with the tumor. Re-operation is mandatory in positive R2 margins and should be considered in R1 when no major morbidity is expected. Affected lymph nodes must be removed but staging lymphadenectomy is not recommended in the absence of radiological evidence (**III,B**). Amputation should be considered when limb preservation is not feasible. However, options for limb-preserving surgery can be discussed with the patient and include preoperative chemotherapy and/or radiotherapy, isolated limb perfusion with tumor necrosis factor-alpha and melphalan (**III,A**) [10], and perioperative regional hyperthermia combined with chemotherapy (see below) (**I,B**).

Radiotherapy combined with surgery improves local control but does not impact overall survival (OS) [11]. Perioperative radiotherapy is the standard treatment for high-grade (G2-3), ≥ 5 cm, and deep tumors (**II,B**). Radiotherapy can be administered pre-operatively or post-operatively, each with its own advantages (**II,B**) [12]. Neoadjuvant radiotherapy allows a lower dose (50 Gy) and a smaller target volume but is associated with increased wound complications. By contrast, later complications (i.e.: fibrosis, edema) are more frequent with adjuvant radiotherapy, which usually involves a higher radiation dose (66 Gy) and tumor volume. The incidence of wound complications may be lower with intensity-modulated radiotherapy (IMRT) compared to traditional external beam radiotherapy (**III,B**) [13]. Combining neoadjuvant radiotherapy and neoadjuvant chemotherapy is feasible and a potential option for selected STS patients, although the added benefit of the combination remains unproven [14] (**II,C**).

Recommendations:

- R0 surgery is the mainstay of treatment (**II,A**).
- Perioperative radiotherapy diminishes the risk of local relapse in high grade, ≥ 5 cm, and deep STS (**II,B**).

#### Adjuvant and neoadjuvant chemotherapy

The use of perioperative chemotherapy with anthracycline plus ifosfamide in surgically resectable STS remains controversial and is not regarded as a standard treatment. However, it may be considered for fit patients with chemo-sensitive, high-risk localized STS subtypes of the limbs and trunk wall, given the potential benefit of improving disease-specific OS (**II,B**). Five cycles of adjuvant epirubicin and ifosfamide constitutes the recommended regimen in high-risk extremity and trunk wall STS (G3, ≥ 5 cm and deep) if post-operatory treatment is indicated. The evidence for this is limited, as it comes from an initially positive small, controlled trial that lost the survival benefit in a longer follow-up and could not be reproduced later in a larger trial due to lower doses [15]. A meta-analysis with 1,953 patients from 18 heterogeneous clinical trials identified a 10% significant decrease in death risk with adjuvant chemotherapy based on the combination of anthracyclines and ifosfamide [16].

The field currently leans more towards the use of neoadjuvant chemotherapy after a phase III, non-inferiority clinical trial showed no differences in OS with three courses of neoadjuvant epirubicin and ifosfamide compared to two additional postoperative courses. However, a proper control arm was lacking (**II,B**) [17]. Neoadjuvant histotype-tailored chemotherapy regimen over the standard chemotherapy regimen shows no benefit and it is not recommended (**I,E**) [18]. The use of regional hyperthermia with systemic chemotherapy could be another option, as the results of a large, randomized phase III study (in patients with G2-3, deep, > 5 cm) had apparently better response rates and a longer disease-free and OS (**I,B**) [19]. Patients with a 10-year predicted OS < 60% should be selected for chemotherapy (**II,B**) [20].

The value of perioperative chemotherapy in low-risk STS, arising in other locations than limbs and trunk-wall, as well as less chemosensitive STS subtypes not included in recently available studies, is not proven. Clinicians are discouraged to use it (**II,D**).

Recommendations:

Neo/adjuvant chemotherapy for at least three cycles of anthracycline and ifosfamide can be considered in high-risk STS-fit patients with chemosensitive subtypes and primary tumors from the extremities and trunk wall (**II,B**).

### Management of advanced and metastatic disease

Up to 50% of STS patients develop metastatic disease, and the current OS of these patients is approximately 20–22 months [21]. Therefore, the inclusion of these patients in clinical trials is highly encouraged in any line of treatment, although earlier lines should be prioritized (**IV,A**) [22].

#### First-line treatment for metastatic disease

Anthracycline-based regimens are the standard first-line treatment for locally advanced and/or metastatic STS with indication for systemic therapy. Six cycles of doxorubicin monotherapy (75 mg/m2 per cycle) is the first-line standard treatment for patients with advanced unresectable STS (**I,A**) [23]. The combination of doxorubicin plus ifosfamide showed a higher response rate and longer PFS in a phase III randomized study, but without OS benefit and increased toxicity [24]. Therefore, it could be only considered in selected patients with sensitive histotypes, in whom tumor shrinkage could bring symptomatic relief or facilitate a potential surgery (**I,B**). The combination of doxorubicin and trabectedin improves OS compared with doxorubicin alone in patients with advanced/metastatic leiomyosarcoma (**II,A**), constituting a potential new standard of care in this setting [25]. Beyond these regimens, other anthracycline combinations have not shown superiority over doxorubicin as a single agent. Doxorubicin plus dacarbazine could be an option in patients with leiomyosarcoma (**IV,B**) [26], as ifosfamide does not show significant activity in this histotype. The combination of gemcitabine and docetaxel failed to show superiority to doxorubicin monotherapy in a randomized phase III study and should not be considered a standard first line unless anthracyclines are contraindicated (**I,D**) [27]. Likewise, high-dose ifosfamide, at 9–14 g/m2, could be an alternative in the first line for patients with advanced synovial sarcoma when anthracyclines are contraindicated (**III,B**) [28]. Weekly paclitaxel is an active regimen in angiosarcoma and could be a first-line option in patients with this histotype (**III,B**) [29]. Finally, a randomized noninferiority trial in elderly (≥ 60 years old) STS advanced/metastatic patients did not show differences between first-line doxorubicin and pazopanib and therefore pazopanib could constitute a less toxic alternative in this population (**I,B**) [30].

For other specific STS subtypes with alternative first-line therapies, please refer to the dedicated section below.

#### Second‐line treatment and beyond

Several treatments have been tested in this setting, and currently there is a lack of direct comparison among them. Thus, the decision is based on histology, toxicity profile, patient preferences, and convenience of the scheme administration (Table 2).

**Table 2:** Systemic agents associated with prospective evidence of activity in selected sarcoma types

Regimen	Sarcoma Type	Line	ORR (%)	PFS (months)	Study Type
Atezolizumab	ASPS	Any	37	20.8	Phase II
Axitinib	SFT	Any	5.9	9.7	Phase II
Cabozantinib / Nivolumab	Angiosarcoma	Prior Taxane	59	9.6	Phase II
Cemiplimab	Secondary Angiosarcoma	1st Line	27.8	3	Phase II
Crizotinib	Inflammatory Myofibroblastic tumor	Any	ALK + : 50 ALK-: 14	N/A	Phase II
Doxorubicin/ Dacarbazine/ Nivolumab	Leiomyosarcoma	1st line	56.2	8.67	Phase Ib
Doxorubicin / Trabectedin	Leiomyosarcoma	1st line	36	12.2	Phase III
Lenvatinib / Eribulin	L-sarcomas	≤ 2 lines	20	8.56	Phase Ib/II
Nilotinib	TGCT	Any	6	77	Phase II
Nirogacestat	Desmoid	Any	41	N/A	Phase III
Paclitaxel / Avelumab	Angiosarcoma	1st line	50	6	Phase II
Pedixartinib	TGCT	Any	39	N/A	Phase III
Regorafenib	1)Leiomyosarcoma	≥ 2nd line	0	3.7	Phase II
	2)Synovial Sarc		8	5.6	
	3) Non-adipocytic		11	2.9	
	4)Non-adipocytic	Prior pazo	0	2.1	Phase II
	5)SFT	Any	37.5	4.7	Phase II
Selumetinib	NF1-related neurofibromas	Any	70	N/A	Phase II
Sorafenib	EHE	Any	13′3	6	Phase II
Vimseltinib	TGCT	> 1 line	40	N/A	Phase III
Tazemetostat	Epithelioid sarc	Any	15	5′5	Phase II
Trametinib	EHE	Any	3′7	10′4	Phase II

ASPS, Alveolar Soft Part Sarcoma; TGCT, Tenosynovial Giant Cell Tumor; SFT, Solitary Fibrous Tumor; STS, Soft Tissue Sarcoma; NF1, Neurofibromatosis Type-1; EHE: Epithelioid hemangioendothelioma; N/A, Not Available

• Ifosfamide: standard dose of ifosfamide at 9 g/m2 can be a second-line option, preferably in patients with sensitive histotypes (**II,B**) [31]. Patients progressing to standard doses of ifosfamide can benefit from high-dose regimens (14 g/m2 as continuous infusion 14 days), especially in patients with synovial sarcoma (**III,B**) [28].

• Gemcitabine-based combinations are well-established second-line options for metastatic STS patients. Gemcitabine (1800 mg/m2 at 10 mg/m2/min) plus DTIC (500 mg/m2) every 14 days showed superior PFS and OS compared to dacarbazine alone along with a good toxicity profile (**II,B**) [32]. This regimen is particularly active in leiomyosarcoma patients, but also in other STS subtypes. Six to eight courses of gemcitabine and docetaxel is another option, with higher toxicity and conflicting results when compared with gemcitabine alone, but showing more activity in leiomyosarcoma and undifferentiated pleomorphic sarcoma patients (**II,C**) [33].

- Trabectedin (1.5 mg/m2 in a 24-h infusion) has a particular PFS benefit in liposarcoma and leiomyosarcoma subpopulations after progression to doxorubicin and ifosfamide (**I,A**) [34]. Low-dose radiotherapy can be safely added to trabectedin in advanced STS if a rapid objective response is needed for a symptomatic or compromising lesion (**III,A**) [35].
- Pazopanib (800 mg daily), a multitargeted tyrosine kinase inhibitor, showed a benefit on PFS versus placebo (4.6 m vs 1.6 m) in a phase III trial in pre-treated patients diagnosed with non-adipocytic STS (**I,A**) [36]. Beyond STS as a whole, pazopanib has also shown activity in selected histotypes (see below).
- Eribulin (1.4 mg/m2 days 1 and 8 every 21 days), has been approved for liposarcoma after progression to anthracycline based on a phase III trial that demonstrated a 7-month gain in OS compared to dacarbazine, but without differences in PFS or RR (**I,A**) [37].

Recommendations:

- Single-agent doxorubicin is the first-line treatment for advanced/metastatic disease (**I,A**). Anthracycline-ifosfamide (STS) can be considered only if significant tumor reduction is clinically needed (**I,B**). Anthracycline-trabectedin can be discussed as a first-line option in leiomyosarcoma (**II,A**), and pazopanib in elderly patients with STS (**I,B**).
- The choice for second and further lines, in the virtual absence of direct comparisons, will be based on histology, toxicity profile, patient preferences, and convenience among these regimens: ifosfamide (**II,B**), gemcitabine-DTIC (**II,B**), gemcitabine-docetaxel (**II,C**), trabectedin (**I,A**), pazopanib (**I,A**), and eribulin (**I,A**).
- It is highly encouraged that advanced/metastatic patients participate in clinical trials from early lines (**IV,A**).

#### Surgery, Radiotherapy, and Other Local Therapies

Occasionally, metastatic STS patients can be managed by combining systemic and/or local approaches. In these cases, the decision-making is complex because it depends on the location and extent of the disease, natural history of the histological subtype and patient fitness, and therefore we recommend having these cases evaluated by a multidisciplinary team in a sarcoma-expert institution (**III,A**).

Oligometastatic disease to the lung, carefully discarding extrapulmonary spread, can be managed with local treatments aiming to improve long-term survival (**IV,B**). A disease-free interval (DFI) of more than one year after primary tumor resection together with low tumor burden (low number of metastases, small size of the largest lesion, and no bilaterality of lung metastases) are the two most relevant predictors for longer outcomes after metastasectomy (**IV,B**) [38]. Video-assisted thoracic surgery (VATS), when feasible, seems not inferior to thoracotomy in expert hands (**IV,B**) [38]. As recurrence after metastasectomy is frequent, repeated pulmonary metastasectomy may be offered to selected patients (**IV,B**) [39]. Stereotactic body radiotherapy (SBRT) constitutes a reasonable and less invasive alternative with excellent rates of local disease control (**IV,B**) [40]. Re-irradiation with SBRT can be an option to be discussed if the patient has already been treated with conventional radiotherapy or SBRT in the ipsilateral lung (**IV,B**). Other local therapies can be considered in experienced centers to treat metastases low-volume metastases, such as radiofrequency, microwave ablation, and cryoablation (**IV,B**). Chemotherapy may be given preferably before local treatment to achieve disease control, although there is a lack of evidence that this sequence improves outcomes (**IV,B**). There is no evidence supporting the use of neoadjuvant or adjuvant treatment for R0 metastatic disease (**IV,D**).

There does not appear to be any added survival benefit of metastasectomy for patients with STS and synchronous metastases or patients with extrapulmonary disease. In the majority of these cases, local treatments may have a palliative role, and systemic treatment is the standard therapy (**IV,C**) [41].

Recommendations:

- Local treatment in advanced STS should be evaluated in sarcoma-expert institutions (**III,A**).
- Surgery and SBRT or the combination of both are the most common approaches (**IV,B**). Palliative chemotherapy can be used before to control the disease (**IV,B**). However, it should never be used with neo/adjuvant intention (**IV,D**).

### Specific adult sarcoma subtypes

The management of these tumors requires specialized evaluation in referral centers with multidisciplinary committees given their rarity and complexity (**III,A**) [6]. Agents with prospective data for specific STS subtypes are summarized in Table 2.

#### Retroperitoneal sarcomas (RPS)

Liposarcoma and leiomyosarcoma are the most common subtypes. Specific nomograms can help in risk assessment and facilitate decision-making. *En bloc* resection of the tumor, including adjacent organs and structures, is the only curative option for RPS (**III,A**) [42]. Negative margins are the main prognostic factor. Preoperative radiotherapy does not improve abdominal recurrence-free survival nor OS and it is not a standard treatment for resectable RPS (**I,D**) [43]. Postoperative RT is discouraged due to the high risk of toxicity (**IV,D**). The value of intraoperative radiotherapy (IORT) remains unproven (**II,C**). Neoadjuvant/adjuvant chemotherapy is not a standard-of-care treatment in RPS and its value in borderline-resectable cases with chemosensitive histologies remain unproven (**IV,C**). The combination of regional hyperthermia and systemic treatment can improve OS and local control in patients with retroperitoneal sarcomas (**I,B**) [19].

Surgery for local recurrences can be offered in selected scenarios based on histology, DFI, morbidity, and potential response to systemic treatment (**IV,B**), which is similar to other STS, also based on histotype.

#### Uterine sarcomas

Uterine sarcomas (US) encompasses various entities such as uterine LMS (uLMS), low- and high-grade endometrial stromal sarcoma (ESS), undifferentiated sarcomas, adenosarcomas, and other rarer subtypes. Localized US are treated with *en bloc* hysterectomy, without morcellation, and with free margins (**II,A**) [44]. Bilateral salpingo-oophorectomy is reasonable in peri/postmenopausal women, although there are no data indicating that improves outcomes. Systemic lymphadenectomy is not recommended [44]. Adjuvant RT can be discussed in a multidisciplinary board in selected high-risk cases (**IV,C**) [45]. The value of adjuvant CT is unproven and generally not recommended (**IV,C**). Adjuvant hormonal therapy in low-grade ESS is not standard, as it has only been evaluated in retrospective studies that suggest decreased relapse (**IV,C**).

Treatment of advanced disease is comparable to that used in the rest of STS and is drawn from studies conducted in this context, and also mainly in uLMS. As special considerations, anti-hormonal therapies constitute a first-line option in low-grade ESS (**IV,B**) and in hormone receptor-positive uLMS, particularly in indolent tumors with low tumor burden (**III,C**). Hyperthermic peritoneal chemotherapy (HIPEC) should only be used only in clinical trials.

#### Desmoid tumor (DT)

DT is a rare monoclonal, fibroblastic proliferation characterized by infiltrative growth and a tendency toward local recurrence but a lack of metastatic potential. Active surveillance (clinical & MRI within 1–2 months, then in 3–6 months intervals) by an experienced multidisciplinary team is the best treatment in asymptomatic patients (**III,A**), especially in unfavorable locations: chest wall, head and neck and upper limbs [46]. Surgery could be considered as the second line, but limited to the abdominal wall, provided the expected surgical morbidity in the rest of locations. Positive microscopic margins can be accepted when function or cosmesis is an issue (**IV,B**). Data for radiotherapy after surgery are limited, but it should be considered when surgery is not an option and medical treatments fail (**IV,B**) [46]. There is randomized data for sorafenib and pazopanib supporting their use as systemic upfront treatment (**II,B**). Chemotherapy, especially doxorubicin or liposomal doxorubicin, is indicated in rapidly growing and/or symptomatic disease (**III,A**). The real activity of other agents (chemotherapy, hormone therapy, nonsteroidal non-inflammatory drugs, interferon) is unknown in the absence of randomized, prospective trials [46]. Nirogacestat, a gamma-secretase inhibitor, has recently shown profound durable benefit in DT in a randomized phase III trial, but is still awaiting financial approval from the health authorities in Spain [47].

#### Alveolar soft part sarcoma (ASPS)

ASPS is a rare STS defined by the ASPSCR1-TFE3 fusion. ASPS usually occurs in adolescents and young adults and has a relatively indolent behavior, but common metastatic spread. ASPS are not sensitive to conventional cytotoxic CT. Atezolizumab has been approved by the FDA after a phase II trial showed 37% ORR and 20.8 months mPFS (**III,A**) [48]. Tyrosine kinase inhibitors (TKIs) with antiangiogenic activity, such as cediranib, sunitinib or pazopanib, have moderate activity in this disease (**III,B**).

#### Dermatofibrosarcoma protuberans (DFSP)

DFSP is a locally-aggressive fibroblastic neoplasm, characterized by dermis location, slow growth, high tendency towards local recurrence, and low metastatic potential. DFSP displays the characteristic fusion gene COL1A1-PDGFB1. In localized disease, Mohs micrographic surgery is the preferred surgical approach (**III,B**). Imatinib is active in DFSP and can be used preoperatively when surgical resection implies excessive functional impairment (**III,B**). Radiotherapy may be considered if positive margins and unfeasible re-excision. High-grade fibro-sarcomatous transformation is seen in 10% of cases and can metastasize. In these cases, the first choice is imatinib (**III,A**). Other TKIs with PDGFRA-inhibitory activity can be used after imatinib failure (**III,B**).

#### Solitary fibrous tumor (SFT)

STF is another rare STS defined by the NAB2-STAT6 fusion, and that typically localizes in the peritoneum, pleura, meninge or limbs. Risk of relapse after surgical resection is predicted by specific criteria. In metastatic malignant SFT, pazopanib is the first choice (**III,B**) [49]. Other antiangiogenic agents, such as sunitinib (**IV,B**) or the combination of temozolomide plus bevacizumab, constitute active options (**IV,B**). Other chemotherapeutic regimens (anthracyclines or DTIC-gemcitabine) can be used as the frontline in dedifferentiated SFT or after TKI failure, albeit the efficacy is unproven (**IV,C**).

#### Other rare STS subtypes

In advanced specific subtypes there is evidence of the activity of several molecular targeted agents based in small retrospective and prospective studies [50]:

- mTOR inhibitors and antiangiogenics in PECOMAS (**IV,B**).
- Trametinib in epithelioid hemangioendothelioma (**III,B**).
- Crizotinib in ALK-translocated inflammatory myofibroblastic tumor (**III,A**).
- Tazemetostat in epithelioid sarcoma (**III,B**).
- NTRK inhibitors (larotrectinib and entrectinib) in NTRK-rearranged STS (**III,A**).

Despite these evidences, only NTRK inhibitors are approved by the health authorities in Spain.

### Follow-up, long-term implications, and survivorship

#### Follow up

Early detection of local or metastatic recurrence might be potentially curable with surgery, SBRT or other ablative techniques. Likewise, the early introduction of systemic treatment for metastatic disease may impact positively on outcomes. However, there is very limited evidence indicating the optimal routine follow-up. Therefore, several aspects should be noted: (1) the individual risk of recurrence (size, grade, histological subtype, and site) must be considered to tailor the follow-up strategy; (2) the timing for relapse varies between high-risk STS, usually within 2–3 years, compared to low-risk and other specific entities that may relapse later; (3) metastatic relapse is most common in the lungs, although some rare subtypes spread to different locations (see above).

In the absence of prospective studies, a sarcoma-expert consensus is followed: after completion of treatment, intermediate-/high-grade patients may be followed every 3–4 months in the first 2–3 years, then twice a year up to the fifth year, and once a year thereafter; low-grade sarcoma patients may be followed every 6 months for the first 5 years, then annually (**IV,C**) (Table 3).

**Table 3:** Recommendations for follow-up

	Recommendation	
Low risk	Local: Physical examination Post-treatment basal CT/MRI/US Distant: Chest X-ray, if M1* nodules chest CT	First 2–3 years: Every 6 m Then annually Every 6–12 months
Intermediate High Risk	Local: Physical examination Post-treatment basal CT/MRI/US Distant: Chest X-ray or chest CT	Every 3–4 months First 2–3 years: Every 3–4 months 3–5 years: every 6 months > 5 years: Annually
Retroperitoneal sarcoma	Abdominopelvic CT Chest X-ray, if M1 nodules chest CT	First 2–3 years: Every 6 months > 3 Years: Annually
Metastatic disease and systemic treatment (outside clinical trial)		Assessment of target lesions (CT, MRI or PET) Individualized follow-up
Educate patients about self-examination		

#### Long-term implications and survivorship

Cancer survivorship requires a comprehensive, multidisciplinary approach to address the long-term effects of cancer and its treatment. Care includes monitoring for recurrence, new primary cancers, and the long-term effects of treatment, such as physical, psychosocial, and immunologic impacts. Specific concerns include cardiotoxicity, especially from anthracyclines and chest radiation, which necessitates ongoing cardiac monitoring. Treatments also risk infertility, prompting pre-treatment counseling and fertility preservation options. Survivors often face mental health challenges, fatigue, and sexual health issues, for which specialist referrals may be needed. Health promotion and surveillance for late effects ensure optimal long-term outcomes for cancer survivors.

Table 4 summarized the main recommendations throughout this guideline.

**Table 4:** List of main recommendations

Recommendations	Level
*Diagnosis, pathology and molecular biology* • Both diagnosis and treatment decision of a clinical suspicion of sarcoma should be conducted in a sarcoma-expert center within a multidisciplinary committee	III, A
*Staging and risk assessment* • CT and/or MRI contrast-enhanced followed by core needle biopsy are the gold standard methods for STS diagnosis	II, A
*Management of local and loco-regional disease* • R0 surgery is the mainstay of treatment (II,A). Perioperative radiotherapy diminishes the risk of local relapse in high grade, ≥ 5 cm, and deep STS	II, B
• Neo/adjuvant chemotherapy for at least three cycles of anthracycline and ifosfamide can be considered in high-risk STS patients with chemosensitive subtypes and primary tumors from the extremities and trunk wall	II, B
*Management of advanced and metastatic disease* • Single-agent doxorubicin is the 1st line in metastatic disease	I,A
• Anthracycline-ifosfamide can be considered only if significant tumor reduction is clinically needed	I,B
• Anthracycline-trabectedin can be discussed as a first-line option in leiomyosarcoma	II,A
• Pazopanib can be considered 1st line in elderly patients	I,B
• The choice for second and further lines, in the virtual absence of direct comparisons, will be based on histology, toxicity profile, patient preferences, and convenience	I,A—II,B
• It is highly encouraged that advanced/metastatic patients participate in clinical trials from early lines	IV,A
• Local treatment in advanced STS should be evaluated in sarcoma-expert institutions	III,A
• Surgery and SBRT or the combination of both are the most common approaches. Palliative chemotherapy can be used before to control the disease	IV,B
